# Evaluation of Antimicrobial Resistance in *Staphylococcus aureus* Isolates by Years

**DOI:** 10.1155/2016/9171395

**Published:** 2016-05-09

**Authors:** Cennet Rağbetli, Mehmet Parlak, Yasemin Bayram, Huseyin Guducuoglu, Nesrin Ceylan

**Affiliations:** ^1^Faculty of Medicine, Department of Medical Microbiology, Yuzuncu Yil University, 65080 Van, Turkey; ^2^Faculty of Medicine, Department of Pediatric Diseases, Yuzuncu Yil University, 65080 Van, Turkey

## Abstract

*Objective*. Recently, community and hospital-acquired infections with* Staphylococcus aureus* have increased and raised antibiotic resistant isolates. In this study, we aimed to evaluate the antibiotic resistance profile of* S. aureus* isolates over several years in various clinical specimens from our hospital.* Materials and Methods*.* S. aureus* strains from 2009 to 2014 were isolated from various clinical samples at Yuzuncu Yil University, Dursun Odabas Medical Center, Microbiology Laboratory, and their antibiotic susceptibility test results were retrospectively investigated. The isolates were identified by conventional methods, and antibiotic susceptibility tests were performed by the Phoenix (Becton Dickinson, USA) automated system method according to Clinical and Laboratory Standards Institute (CLSI) standards.* Results*. A total of 1,116* S. aureus* isolates were produced and methicillin-resistant* S. aureus* (MRSA) to 21% of all* S. aureus* isolates between 2009 and 2014. According to the results of susceptibility tests of all isolates of* S. aureus*, they have been identified as sensitive to vancomycin, daptomycin, linezolid, and levofloxacin. While the resistance rates to nitrofurantoin, quinupristin-dalfopristin, and trimethoprim-sulfamethoxazole were determined as 0.3%, 2.4%, and 6%, respectively, resistance rates to penicillin, erythromycin, rifampicin, gentamicin, and clindamycin were determined as 100%, 18%, 14%, 14%, and 11%, respectively. The highest percentage of methicillin resistance was determined as 30% in 2009, and the resistance was determined to have decreased in subsequent years (20%, 16%, 13%, 19%, and 21%) (*p* < 0.001).* Conclusion*. Currently, retrospective evaluations of causes of nosocomial infection should be done periodically. We think that any alteration of resistance over the years has to be identified, and all centers must determine their own resistance profiles, in order to guide empirical therapies. Reducing the rate of antibiotic resistance will contribute to reducing the cost of treatment.

## 1. Introduction

*Staphylococcus aureus*, involved in the Micrococcaceae family, is a Gram-positive, catalase, and coagulase-positive bacteria.* Staphylococcus *spp. are common in nature and human flora. In spite of being asporous, it is among the bacteria that are most resistant to the external environment and disinfectant agents. These resistant isolates can cause various clinical situations varying between superficial infections and serious life-threatening infections. Therefore,* S. aureus* should be frequently isolated from the community and hospital-acquired infections [1–3].

Hospital-acquired methicillin-resistant* S. aureus* (MRSA) infections are the factors held most responsible for mortality and morbidity in Turkey and the world. MRSA can easily be spread from patient to patient through the hands of the staff and can lead to frequent epidemics [4, 5]. MRSA colonization is common in hospitals. MRSA colonization rates vary between 10% and 20% [6]. Hand hygiene and isolation measures can be preventive for MRSA. These measures have been shown to reduce hospital-acquired MRSA infections. However, effort has been expended for a long time toward preventing hospital-acquired MRSA infections, and effective infection control measures have been put into practice [7, 8]. This study aims to retrospectively determine the resistance rates of* S. aureus* strains, isolated from clinical samples in our hospital, against methicillin and other antibiotics and to set out the changes in detail.

## 2. Materials and Methods

The antibiotic resistance rates of* S. aureus* strains isolated from various clinical samples at Yuzuncu Yil University, Dursun Odabas Medical Center, Microbiology Laboratory, between January 2009 and May 2014, were evaluated retrospectively. Culture samples were taken from the abscess and the wounds using needle aspiration and swab technique. Clinical samples were cultivated to a 5% sheep blood agar medium and were incubated at 37°C for 18–24 hours. Isolates produced on sheep blood agar at the end of this period were defined by colony morphology, Gram stain, catalase test, and coagulation test. Verification and antibiograms of all isolates were conducted using Phoenix (Becton Dickinson, USA) automated systems. These systems use microdilution method for antibiotic susceptibility tests. The interpretations of antibiotic resistance for all years were based on CLSI criteria [9]. Specific antibiotics were tested in all isolates according to the CLSI guidelines. Statistically, *Z*-test and rate comparison were made to determine the resistance rates against methicillin and other antibiotics according to year.

## 3. Results

During the study, a total of 1,116* S. aureus* isolates from various clinics were collected. Of these isolates, 339 (30.4%) were obtained from wounds, 286 (25.6%) from ears, 141 (12.6%) from blood, 90 (8.1%) from tracheostomy material, 85 (7.6%) from urine, 83 (7.4%) from abscesses, and 92 (8.2%) from other clinical samples. The distribution of isolated strains according to the clinic and the type of sample is given in Tables 1 and 2.

According to the susceptibility test results done for all years, all* S. aureus* isolates were identified as sensitive against vancomycin, daptomycin, linezolid, and levofloxacin. Resistance rates against nitrofurantoin, quinupristin-dalfopristin, and trimethoprim-sulfamethoxazole were determined as 0.3%, 2.4%, and 6.1%, respectively. Penicillin (1,033/1,033; 100%), erythromycin (183/1,034; 17.7%), rifampicin (156/1,116; 14%), gentamicin (145/1,116; 13.8%), and clindamycin (108/977; 11.1%) were determined to be antibiotics with the highest resistance (Table 3). The methicillin resistance rate of* S. aureus* strains over all the years was determined to be 20.1% in total. Methicillin resistance was highest in 2009 and decreased in subsequent years. High rate of MRSA in 2009 was determined to be statistically significant compared to the rates during 2010–2013 (*p* < 0.01); no statistically significant difference was found in MRSA isolates between 2009 and 2014 (*p* > 0.05).

## 4. Discussion

*Staphylococcus* has emerged as a worldwide life-threatening hospital- and community-acquired infection factor. The antimicrobial resistance problem in MRSA isolates in hospital infections is accompanied by high morbidity and mortality. The prevalence of MRSA infections can vary from country to country and between hospitals, and it also varies between different units of the same hospital [7, 8, 10].

In a surveillance study including 495 centers from 26 countries in Europe, while the MRSA prevalence of the Northern European countries was below 1%, it was determined to have reached to 50% in Southwest European countries and the United States [11–13]. In studies conducted in Turkey Cıtak and Karacocuk [14] determined an MRSA resistance rate of 40% in hospital-acquired infections and of 31% in community-acquired infections. Yilmaz et al. [15] determined a methicillin resistance of 61.1% for hospital-acquired isolates and of 6.7% for community-acquired isolates between 2007 and 2010. Haznedaroglu et al. [16], in their three-year surveillance study covering the years 2006, 2007, and 2008, determined an MRSA rate at 56.6%, 39.3%, and 42.0%, respectively. Aydin et al. [17] reported the MRSA rate as 10.9%, and Ozkalp and Baybek [18] reported it as 20.6%. In our study, the MRSA rates by year were determined as, respectively, 30%, 19.6%, 16.4%, 12.5%, 18.5%, and 20.8% (2009–2014). High MRSA rates in 2009 were statistically significant compared to those of other years. We consider this case as a positive reflection of the decrease in MRSA, proper basic infection control measures, regular implementation of MRSA screening, and decolonization for individuals in high-risk group and successful MRSA education programs.

Glycopeptide antibiotics such as vancomycin are generally used in treatment against MRSA strains in Turkey and in the rest of the world. But the widespread use of vancomycin after methicillin-resistant* S. aureus* MRSA infections has caused a decrease in vancomycin sensitivity in many countries. Following the identification of vancomycin intermediate-resistant* S. aureus* (VISA) strains for the first time in Japan in 1997, glycopeptide-resistant staphylococci strains have led to concern in terms of hospital infections. A new vancomycin resistance defined as heteroresistant VISA (hVISA) was also identified in the same year as the VISA strains [19, 20]. VISA and hVISA strains have also been reported in Turkey since 2005 [21]. Vancomycin resistance has not been detected in several studies conducted both in Turkey and in Europe [10, 18, 22]. In this study, while all the isolated* S. aureus* strains were determined by vancomycin sensitive, no VISA and hVISA were detected. Accordingly, vancomycin is among the antibiotics appropriate to use in the presence of MRSA. For linezolid, an oxazolidinone group drug, Jones et al., in their study of 18,527 isolates carried out in 26 European countries, found* S. aureus* resistance rates to be 0.1% [23]. Resistance has not been reported in various in vitro susceptibility studies carried out for linezolid in MRSA isolates in Turkey [24]. Quinupristin/dalfopristin is an effective agent in severe infections caused by particularly life-threatening multidrug-resistant* S. aureus*. Success treatment rate of 71.1% has been reported for these infections [25]. In studies performed in Turkey, while Baysallar et al. [26] reported a quinupristin/dalfopristin resistance for MRSA of 1%, Tunger et al. [27] reported it as 2.3%. Daptomycin, one of the new antimicrobial agents for the treatment of the infections caused by resistant Gram-positive species, is highly effective in the treatment of complicated skin and soft tissue infections. In studies conducted in Turkey for* S. aureus* strains, daptomycin resistance was not observed [28, 29]. In our study, consistent with the literature, no resistance was observed in any strains against linezolid or daptomycin, and the quinupristin/dalfopristin resistance rate was determined to be 2.4%, averaged over all years.

Norfloxacin, quinolones, and trimethoprim are among the antibiotics included in group U (for urine samples) and group C (additional limited notification) in the CLSI guidelines [9]. Ozkalp and Baybek [18] found that* S. aureus* strains showed resistance at the rate of 29.8% to trimethoprim-sulfamethoxazole. Aydin et al. [17] determined that the strains were resistant to trimethoprim-sulfamethoxazole at a rate of 15.8% and were resistant at a rate of 7.3% to ciprofloxacin. Eksi et al. [30] determined low rates of resistance in methicillin-sensitive* S. aureus* (MSSA) strains, including a 2.4% rate for ciprofloxacin and a 1.6% rate for nitrofurantoin, and in MRSA strains, they determined low rates of resistance including 90.8% to ciprofloxacin and 0.8% to nitrofurantoin. In our study, while resistance rates for norfloxacin, trimethoprim-sulfamethoxazole, and nitrofurantoin were determined as 10.3%, 6.1%, and 0.3%, respectively, levofloxacin-resistant strains were not detected.

Considering the studies examining antibiotic resistance in* S. aureus* strains, Ozkalp and Baybek [18] found that the strains showed resistance to penicillin G at the rate of 85.8% and to erythromycin at the rate of 87.2%. Aydin et al. [17] determined resistance to penicillin at the rate of 92.3%, at 21.5% to erythromycin, and at 14.8% to clindamycin. Eksi et al. [30], in their study, determined resistance to penicillin at the rate of 87.8%, at 6.5% to rifampin, and at 1.6% to gentamicin in MSSA strains. In MRSA strains, they determined resistance at high rates such as 80.8% to rifampin and 85% to gentamicin. Gursoy et al. [22] determined resistance at the rate of 94% to penicillin, 29% to erythromycin, 19% to clindamycin, 26% to gentamicin, 29% to rifampicin, and 34% to tetracycline in* S. aureus* strains isolated from blood cultures. In our study, resistance to penicillin was determined at the rate of 100% due to beta-lactamase production. The resistance ratios obtained for erythromycin, rifampin, gentamicin, clindamycin, and tetracycline were determined to be close to or lower than the rates reported in the literature.

In conclusion, the most effective antibiotics in* S. aureus* strains were identified as vancomycin, linezolid, levofloxacin, and daptomycin. Surveillance studies need to be carried out periodically in every hospital to engage in an effective fight against MRSA-based hospital infections and to reduce resistance rates. Improper use of antibiotics in choosing empirical treatment will prevent the reduction of and will enable multiresistant bacteria infections.

## Figures and Tables

**Table 1:** Distributions of *S. aureus* strains according to the clinics.

Name of the clinic	*n*	%
Ear, nose, and throat	318	28.5
Pediatrics	213	19.1
Orthopedics and traumatology	139	12.5
Dermatology	117	10.5
Internal diseases	66	5.9
Intensive care	63	5.6
Infectious diseases	36	3.2
Pulmonology	25	2.2
Others	139	12.5

*Total*	*1116*	*100*

**Table 2:** Distributions of *S. aureus* strains according to sample types.

Sample types	*n*	%
Wound	339	30.4
Ear	286	25.6
Blood	141	12.6
Tracheostomy material	90	8.1
Urine	85	7.6
Abscess	83	7.4
Others^*∗*^	92	8.2

*Total*	*1116*	*100*

^*∗*^Cerebrospinal fluid (CSF), synovia, bone marrow, conjunctiva, cornea, paracentesis, and empyema.

**Table 3:** Antibiotic resistance rates of isolated *S. aureus* strains by years (%).

Name of antibiotic	Resistance rates by years (%)						Total
	2009	2010	2011	2012	2013	2014	
MRSA	30	19.6	16.4	12.5	18.5	20.8	20.1
Penicillin G	100	100	100	100	100	100	100.0
Erythromycin	30.2	11.3	15	15.7	15.1	17.6	17.7
Rifampin	26	17	9.7	5.6	9.6	10.4	14.0
Gentamicin	26	16.1	10.4	5.6	6.6	7.8	13.0
Clindamycin	11.8	4.5	11.7	11.9	14.2	13.5	11.1
Norfloxacin	TE	17.8	8.1	7.7	10.6	8	10.3
Tetracycline	0	9.8	12.7	13.2	12.4	13	10.1
TMP-SXT	5.8	4.8	5.4	8.5	7	5.2	6.1
Q/D	TE	2.2	1.2	1.4	4.1	0	2.4
Nitrofurantoin	TE	0	0	0	0	2.7	0.3
Daptomycin	TE	0	0	0	0	0	0
Levofloxacin	0	0	0	0	0	0	0
Linezolid	0	0	0	0	0	0	0
Vancomycin	0	0	0	0	0	0	0

MRSA: methicillin-resistant *S. aureus*, TE: not tested, Q/D: quinupristin/dalfopristin, TMPSXT: trimethoprim-sulfamethoxazole.
